# Effects of different antibiotic classes on airway bacteria in stable COPD using culture and molecular techniques: a randomised controlled trial

**DOI:** 10.1136/thoraxjnl-2015-207194

**Published:** 2015-07-15

**Authors:** Simon E Brill, Martin Law, Ethaar El-Emir, James P Allinson, Phillip James, Victoria Maddox, Gavin C Donaldson, Timothy D McHugh, William O Cookson, Miriam F Moffatt, Irwin Nazareth, John R Hurst, Peter M A Calverley, Michael J Sweeting, Jadwiga A Wedzicha

**Affiliations:** 1 National Heart and Lung Institute, Imperial College London, London, UK; 2 Medical Research Council Biostatistics Unit Hub for Trials Methodology Research, Cambridge, UK; 3 Centre for Clinical Microbiology, University College London, London, UK; 4 Department of Primary Care and Population Sciences, University College London, London, UK; 5 Centre for Respiratory Medicine, University College London, London, UK; 6 School of Aging and Chronic Disease, University of Liverpool, Liverpool, UK; 7 Department of Public Health and Primary Care, University of Cambridge, Cambridge, UK

**Keywords:** COPD Exacerbations, COPD Pathology, Respiratory Infection

## Abstract

**Background:**

Long-term antibiotic therapy is used to prevent exacerbations of COPD but there is uncertainty over whether this reduces airway bacteria. The optimum antibiotic choice remains unknown. We conducted an exploratory trial in stable patients with COPD comparing three antibiotic regimens against placebo.

**Methods:**

This was a single-centre, single-blind, randomised placebo-controlled trial. Patients aged ≥45 years with COPD, FEV_1_<80% predicted and chronic productive cough were randomised to receive either moxifloxacin 400 mg daily for 5 days every 4 weeks, doxycycline 100 mg/day, azithromycin 250 mg 3 times a week or one placebo tablet daily for 13 weeks. The primary outcome was the change in total cultured bacterial load in sputum from baseline; secondary outcomes included bacterial load by 16S quantitative PCR (qPCR), sputum inflammation and antibiotic resistance.

**Results:**

99 patients were randomised; 86 completed follow-up, were able to expectorate sputum and were analysed. After adjustment, there was a non-significant reduction in bacterial load of 0.42 log_10_ cfu/mL (95% CI −0.08 to 0.91, p=0.10) with moxifloxacin, 0.11 (−0.33 to 0.55, p=0.62) with doxycycline and 0.08 (−0.38 to 0.54, p=0.73) with azithromycin from placebo, respectively. There were also no significant changes in bacterial load measured by 16S qPCR or in airway inflammation. More treatment-related adverse events occurred with moxifloxacin. Of note, mean inhibitory concentrations of cultured isolates increased by at least three times over placebo in all treatment arms.

**Conclusions:**

Total airway bacterial load did not decrease significantly after 3 months of antibiotic therapy. Large increases in antibiotic resistance were seen in all treatment groups and this has important implications for future studies.

**Trial registration number:**

clinicaltrials.gov (NCT01398072).

**Key messages**

**What is the key question?**

- How do three different antibiotics (moxifloxacin, doxycycline and azithromycin) compare in their effects on total airway bacterial load in stable COPD?

**What is the bottom line?**

- Airway bacterial load was similar to placebo after treatment with all three antibiotics, although increases in antimicrobial resistance were noted in all treatment arms.

**Why read on?**

- This is the first trial to directly compare the effects of different antibiotic classes on airway bacterial load in stable COPD.

## Introduction

COPD exacerbations are important events associated with poorer health status,^1^ lung function decline^2^ and mortality.^3^ Their prevention is an important goal of COPD treatment^4^ ^5^ which is not currently met by existing inhaled therapies.^6^ ^7^

Exacerbation frequency is linked to airway bacterial colonisation during the stable state,^8^ and long-term antibiotic therapy has been proposed to prevent exacerbations. The best evidence for this comes from the macrolide antibiotics, with both erythromycin^9^ and azithromycin^10^ shown to significantly reduce exacerbation frequency although at the expense of auditory decrements and a possible increase in cardiovascular risk.^11^ There is less evidence for other antibiotic classes in stable COPD, although the fluoroquinolone moxifloxacin has shown some efficacy when used in a pulsed dosing regimen,^12^ and with no comparative studies there are few data to inform the optimum antibiotic choice. There are also growing concerns regarding the development of antibiotic resistance in airway bacteria.^13^

Although the presumptive mode of action of antibiotics is via reducing airway bacterial load, no trials to date have examined this in stable COPD. Furthermore, molecular techniques are now important tools in accurately quantifying the airway microbiome^14^ and may be able to detect more subtle changes with therapy than traditional culture-based methods.

For the first time, we aimed to directly compare the effects of different antibiotics on airway bacterial load in patients with stable COPD, measured both by culture and 16S quantitative PCR (qPCR). We also examined important secondary clinical endpoints, bacterial resistance and changes in airway inflammation.

## Methods

### Study design

This was an exploratory 13-week, single-centre, single-blind, placebo controlled, randomised controlled trial conducted at the Royal Free Hospital (London, UK). We compared the efficacy of pulsed moxifloxacin 400 mg daily for 5 days every 4 weeks, doxycycline 100 mg daily, azithromycin 250 mg three times per week, or placebo one tablet daily, at reducing bacterial numbers in spontaneously expectorated sputum after 13 weeks of treatment.

Research ethics approval was granted by King's College Regional Ethics Committee (reference 11/LO/0932).

### Participants

Subjects were recruited from primary and secondary care; further details can be found in the online supplementary data.

Screening and all study visits took place in the outpatient department at the Royal Free Hospital, London. Sputum processing and culture, sensitivity testing and inflammatory marker analysis were conducted in the laboratories of the Centre for Respiratory Medicine and the Centre for Clinical Microbiology, Royal Free Campus, University College London. DNA extraction and 16S qPCR were conducted in the laboratories of the National Heart and Lung Institute, Imperial College London.

### Inclusion and exclusion criteria

Stable patients aged ≥45 years with chronic bronchitis (self-reported sputum expectoration on most days when clinically stable) and spirometrically confirmed COPD (defined by FEV_1_<80% predicted, FEV_1_ to FVC ratio <0.7 and a history of smoking) were included. Patients who reported either treatment for an exacerbation or an episode of symptom worsening in the 4 weeks prior to screening, or were unable to enrol for safety reasons, were excluded. Detailed inclusion and exclusion criteria can be found in the online supplementary data.

### Screening and run-in period

After clinical assessment, spirometry was performed in accordance with American Thoracic Society/European Respiratory Society guidance^15^ using a Vitalograph Gold Standard spirometer (Vitalograph, Maids Morton, UK). Spontaneously expectorated sputum was collected for analysis prior to randomisation. Patients also completed the St George's Respiratory Questionnaire (SGRQ) to assess health status. Patients received diary cards and recorded any worsening of their respiratory symptoms each day for a 1-week run-in period; these have previously been shown to be a reliable index of exacerbation.^16^

### Randomisation and masking

Patients returned to the clinic 1 week after screening. The diary cards and screening results were checked and details sought regarding any adverse events. If the subject remained eligible and clinically stable after reassessment, and had been able to provide a sputum sample for analysis by the second visit, then internet randomisation into groups of 1:1:1:1 was performed using a computer-generated permuted block system of variable sizes (Sealed Envelope, UK). Patients remained blinded to treatment allocation.

### Follow-up of patients

Patients were telephoned by the study doctor during weeks 5 and 9 of treatment and any adverse events or exacerbations recorded. Patients who suffered a COPD exacerbation during the trial were treated either by their treating physician or using self-administered treatment as appropriate; exacerbation treatment during the study was not standardised. To avoid drug interactions, the study medication was temporarily stopped if the patient took other antibiotics for any reason. Details of exacerbations were recorded on the daily diary cards and the study team notified.

Patients attended the clinic at the end of 13 weeks of treatment. A further spontaneously expectorated sputum sample was collected and the SGRQ administered, diary cards collected and any exacerbations or adverse events recorded. Unused medication was collected and pill counts performed for adherence analysis.

### Outcome measures

The primary outcome was the change in sputum bacterial load, as assessed by quantitative culture. Prespecified secondary outcomes were changes in resistance to the three tested antibiotics, changes in FEV_1_, adherence to therapy, health status as measured by total SGRQ scores and adverse events. Further exploratory outcomes assessed changes in sputum bacterial load as assessed by 16S rRNA gene targeted qPCR and changes in sputum inflammation.

### Laboratory analysis of sputum

A minimum of 0.25 g spontaneously expectorated sputum was analysed using a modification of the method described by Pye *et al*.^17^

Mean inhibitory concentrations (MICs) were established for each isolate against each of the three antibiotics by Etest (bioMérieux UK, Basingstoke, UK) and isolates were defined as sensitive, intermediate or resistant where breakpoints were available.

DNA was isolated from sputum and qPCR for 16S bacterial ribosomal RNA gene carried out using a ViiA 7 real-time PCR system (Life Technologies, Paisley, UK).

Sputum supernatant was batch analysed for the cytokines interleukin (IL)-6, IL-8 and IL-1β using commercial high-sensitivity sandwich ELISA kits (RD Systems, Abingdon, UK). The lower limits of detection were 0.70, 3.5 and <1.0 pg/mL, respectively.

Further detail on these methods is included in the online supplementary data.

### Statistical analysis

Pre-existing comparative data showing the effect of antibiotics on sputum bacterial numbers using quantitative culture methods were unavailable. Using PCR analysis, data from within our group showed that, in patients demonstrating bacterial colonisation on ≥2 occasions, mean (SD) sputum bacterial load when stable was 6.89 (1.23) log_10_ cfu/mL, falling to 5.01 (3.71) after antibiotic treatment at exacerbation. Assuming a correlation of 0.5 between the baseline and 3-month follow-up measurements, we calculated that 44 patients in each of the four groups would be sufficient to have a 90% chance of detecting, as significant at the 5% level, a difference between untreated (placebo) and post-treatment bacterial load of 1.88 log_10_ cfu/mL.

Analysis was by intention to treat. The primary endpoint of total bacterial numbers in sputum (the sum of the bacterial load for each isolate cultured from a sample) was analysed using multiple regression after log_10_ transformation to assess the treatment effect for each antibiotic compared with placebo. Where growth was below the limit of detection (6×10^6^ cfu/mL), patients were treated as having a left-censored outcome and a parametric survival regression model was used. Spirometry, health status (SGRQ) and adherence to therapy were modelled using linear, Poisson or logistic regression as appropriate. Antibiotic resistance was modelled using a linear mixed effects model for log(MIC) to account for multiple detected isolates within each individual; a generalised mixed effects model was used to analyse resistance as a binary outcome. All analyses were adjusted for baseline values, with multivariable analyses further adjusted for age, sex, current smoking status, FEV_1_% predicted and previous exacerbation history. Exacerbations during the study period were defined using diary card criteria as previously reported^18^ as well as patient reporting to clinical health professionals or the study team, with the final decision made by consensus. A further methodological analysis compared the measurement techniques of quantitative culture and 16S qPCR using the Bland–Altman method^19^ for all samples where both results were available.

## Results

### Patient characteristics at recruitment

**Figure 1:**
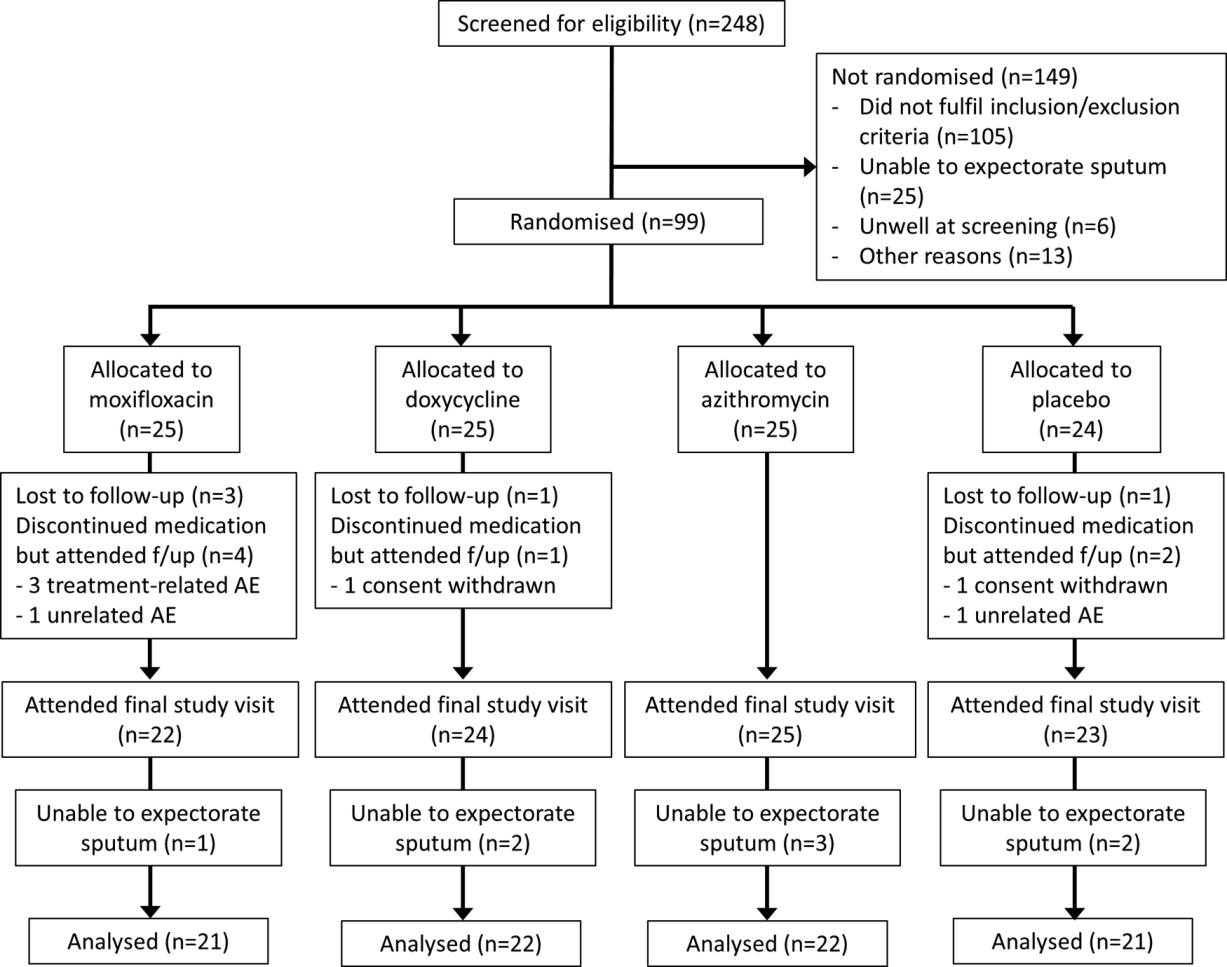
CONSORT diagram for this study showing screening, patient recruitment and data flow for the primary endpoint.

Two hundred and forty-eight patients were screened between February 2012 and May 2013; 99 were randomised, and sputum was analysed for the primary endpoint from 86. Figure 1 shows the screening, randomisation and follow-up of patients. Patient characteristics at baseline are summarised in table 1. This cohort of patients was representative of the population with moderate–severe COPD, with a male preponderance, overall mean age of 69·5 years and mean FEV_1_ 50·5% predicted.

**Table 1:** Patient characteristics at baseline (mean (SD) unless stated)

Treatment group	Moxifloxacin	Doxycycline	Azithromycin	Placebo
Total (n)	25	25	25	24
Gender (n, % male)	17 (68)	18 (72)	16 (64)	18 (75)
Age (years)	70.9 (8.2)	70.4 (7.0)	67.9 (8.6)	68.7 (9.8)
BMI (kg/m^2^)	26.3 (5.2)	28.4 (6.4)	26.6 (6.9)	26.9 (4.9)
Current smoker, n (%)	16 (64)	10 (40)	7 (28)	8 (33)
Pack-years	53 (27)	52 (50)	51 (25)	56 (50)
Number of exacerbations in previous year*	2.5 (2.1)	2.1 (1.7)	2.8 (4.0)	1.5 (1.4)
Inhaled corticosteroid use, n (%)	21 (84)	19 (76)	18 (72)	13 (57)
Bacterial load, log_10_ cfu/mL†	8.3 (0.8)	8.1 (0.7)	8.1 (0.8)	7.8 (0.7)
Bacterial load, log_10_ 16S copies/g sputum	9.4 (0.8)	9.3 (0.73)	9.0 (0.6)	9.1 (0.8)
FEV_1_ (L)	1.4 (0.5)	1.5 (0.5)	1.2 (0.5)	1.5 (0.6)
FEV_1_:FVC	0.51 (0.10)	0.51 (0.13)	0.45 (0.12)	0.51 (0.12)
FEV_1_, % predicted	52 (13)	53 (14)	44 (17)	53 (13)
FVC (L)	2.8 (1.1)	3.0 (1.1)	2.7 (0.7)	3.0 (1.0)
SGRQ: total score	51 (14)	47 (16)	48 (18)	46 (19)
SGRQ: symptom score	64 (16)	62 (24)	59 (18)	55 (19)
SGRQ: activity score	67 (21)	62 (19)	66 (25)	61 (24)
SGRQ: impact score	37 (12)	35 (16)	35 (18)	34 (20)
Il-1β, log_10_ pg/mL	2.3 (0.6)	1.9 (0.7)	2.2 (0.8)	2.1 (0.7)
IL-6, log_10_ pg/mL	1.9 (0.7)	1.5 (0.8)	1.8 (0.8)	1.6 (0.6)
IL-8, log_10_ pg/mL	4.3 (0.7)	3.9 (0.9)	4.1 (0.7)	3.8 (0.8)

*Self-reported exacerbation frequency.

†For those patients with culture results above the threshold of detection.

BMI, body mass index; IL, interleukin; SGRQ, St George's Respiratory Questionnaire.

### Primary endpoint: bacterial numbers

In an analysis of bacterial load, adjusted only for baseline values, there was a mean change of −0·32 log_10_ cfu/mL (95% CI −0.81 to 0.17, p=0.20) from placebo in the moxifloxacin arm, −0·05 (95% CI −0.50 to 0.40, p=0.82) in the doxycycline arm and −0.17 (95% CI −0.62 to 0.29, p=0.47) in the azithromycin arm. The largest change in bacterial numbers was therefore seen with moxifloxacin, equivalent to a 62% reduction, although this was not statistically significant at the 5% level. The smallest change was seen with azithromycin. These results are summarised in table 2.

**Table 2:** Primary and secondary outcome measures for this study

Drug	Estimated change from placebo	
	Baseline adjusted estimate (95% CI), p value	Fully adjusted* estimate (95% CI), p value
Bacterial load by quantitative culture (log_10 _cfu/mL)		
Moxifloxacin	−0.32 (−0.81 to 0.17) p=0.20	−0.42 (−0.91 to 0.08) p=0.10
Doxycycline	−0.05 (−0.50 to 0.40), p=0.82	−0.11 (−0.55 to 0.33), p=0.62
Azithromycin	−0.17 (−0.62 to 0.29), p=0.47	−0.08 (−0.54 to 0.38), p=0.73
Bacterial load by 16S qPCR (log_10_ copies/g of sputum)		
Moxifloxacin	0.14 (−0.42 to 0.69), p=0.63	0.30 (−0.30 to 0.89), p=0.33
Doxycycline	0.06 (−0.49 to 0.60), p=0.84	0.16 (−0.40 to 0.72), p=0.58
Azithromycin	0.28 (−0.27 to 0.83), p=0.33	0.32 (−0.24 to 0.88), p=0.27
FEV_1_, mL		
Moxifloxacin	39 (−84 to 161), p=0.54	58 (−74 to 190), p=0.39
Doxycycline	31 (−89 to 151), p=0.61	39 (−85 to 162), p=0.54
Azithromycin	1 (−123 to 124), p=0.99	−1 (−126 to 125), p=0.99
SGRQ total score		
Moxifloxacin	−2.25 (−8.60 to 4.09), p=0.49	−1.88 (−8.59 to 4.84), p=0.59
Doxycycline	0.88 (−5.30 to 7.06), p=0.78	1.02 (−5.28 to 7.31), p=0.75
Azithromycin	−2.35 (−8.44 to 3.73), p=0.45	−2.29 (−8.43 to 3.86), p=0.47
Adherence to treatment. OR (95% CI)		
Moxifloxacin	0.89 (0.05 to 15.09), p=0.94	0.74 (0.04 to 15.61), p=0.85
Doxycycline	0.64 (0.05 to 9.02), p=0.74	0.60 (0.04 to 9.68), p=0.72
Azithromycin	1.34 (0.07 to 26.06), p=0.84	2.42 (0.09 to 63.28), p=0.60
Exacerbation frequency. Relative risk (95% CI)		
Moxifloxacin	1.36 (0.62 to 2.97), p=0.44	1.38 (0.62 to 3.10), p=0.43
Doxycycline	2.05 (0.98 to 4.29), p=0.06	2.07 (0.99 to 4.35), p=0.05
Azithromycin	0.83 (0.35 to 1.93), p=0.66	0.72 (0.30 to 1.71), p=0.45
*Antibiotic resistance testing*		
	Factor change in MIC	
Moxifloxacin	4.82 (1.44 to 16.19), p=0.01	
Doxycycline	3.74 (1.46 to 9.58), p=0.01	
Azithromycin	6.23 (1.66 to 23.35), p=0.01	
	OR for resistant isolates	
Moxifloxacin	2.03 (0.36 to 11.54, p=0.42	
Doxycycline	5.77 (1.40 to 23.74, p=0·02)	
Azithromycin	2.42 (0.61 to 9·62, p=0.21)	
*Sputum inflammatory markers*		
IL-1β (log_10 _pg/mL)		
Moxifloxacin	−0.25 (−0.63 to 0.14), p=0.21	−0.15 (−0.56 to 0.26), p=0.47
Doxycycline	−0.10 (−0048 to 0.28), p=0.61	−0.03 (−0.43 to 0.37), p=0.88
Azithromycin	0.04 (−0.35 to 0.43), p=0.86	0.09 (−0.31 to 0.49), p=0.66
IL-6 (log_10_ pg/mL)		
Moxifloxacin	−0.16 (−0.50 to 0.18), p=0.37	−0.22 (−0.59 to 0.16), p=0.27
Doxycycline	−0.21 (−0.56 to 0.13), p=0.22	−0.23 (−0.58 to 0.13), p=0.25
Azithromycin	−0.19 (−0.43 to 0.27), p=0.65	−0.06 (−0.42 to 0.30), p=0.77
IL-8 (log_10_ pg/mL)		
Moxifloxacin	−0.26 (−0.72 to 0.19), p=0.26	−0.29 (−0.78 to 0.19), p=0.24
Doxycycline	−0.11 (−0.55 to 0.34), p=0.64	−0.08 (−0.54 to 0.38), p=0.74
Azithromycin	0.00 (−0.46 to 0.46), p=1.00	0.02 (−0.45 to 0.49), p=0.94

*The results in the right hand column were adjusted for age, sex, smoking status, FEV_1_% predicted and prior exacerbation history. p Values were calculated using the Wald test.

IL, interleukin; MIC, mean inhibitory concentration; qPCR, quantitative PCR; SGRQ, St George's Respiratory Questionnaire.

**Figure 2:**
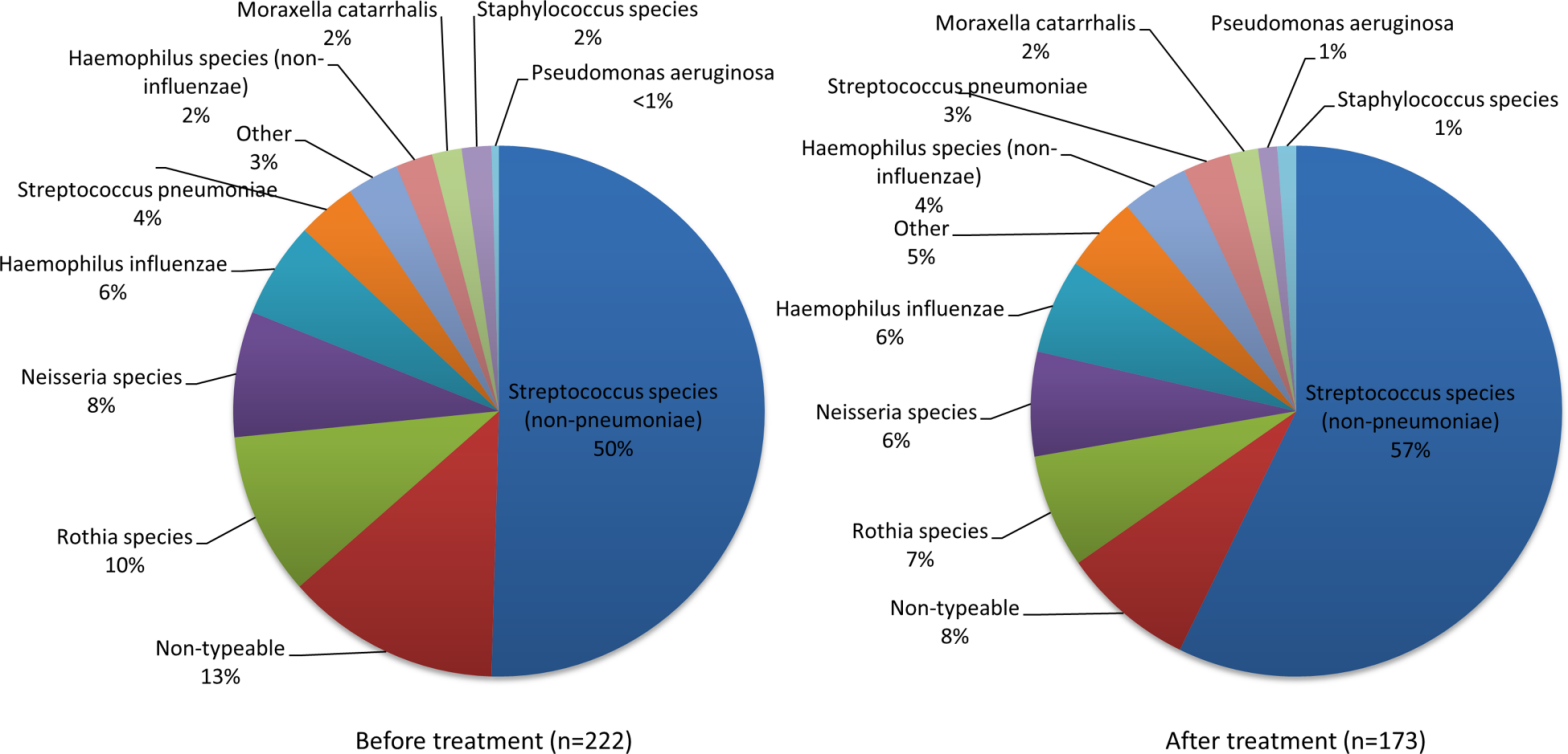
Species breakdown of all cultured isolates (n=395) before and after treatment.

A total of 395 isolates were cultured: 222 at study start and 173 after treatment. The most common isolates were *Streptococcus* spp other than *Streptococcus pneumoniae*. Figure 2 shows the species breakdown of these isolates.

### Secondary and exploratory endpoints

#### Bacterial numbers by 16S qPCR

One hundred and forty-two paired samples from 71 patients were available for this analysis. As with quantitative culture, there were no significant changes in overall bacterial load compared with placebo in any of the antibiotic treatment arms (table 2).

#### Sputum inflammatory markers

These were also measured in the subset of patients where extra paired sputum samples were available (n=71). No significant changes were seen in any of the three measured cytokines IL-6, IL-8 and IL-1β in any of the antibiotic treatment arms compared with placebo (table 2).

#### Bacterial resistance

**Figure 3:**
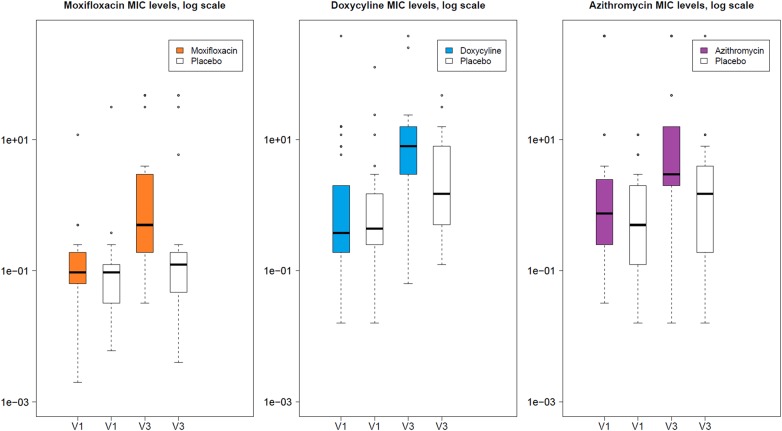
Boxplots for each treatment arm showing mean inhibitory concentrations (MICs) against that antibiotic compared with placebo before and after 3 months of treatment. Note that MICs for all detected isolates are shown, and the number of isolates before and after treatment is not necessarily comparable.

Two hundred and forty-three isolates from 69 patients where ≥1 isolate was cultured both pre and post treatment were tested for bacterial resistance. There were measurable increases in the degree of antibiotic resistance of isolates in all three antibiotic arms. After adjusting for baseline MIC and whether the isolate was a species known to be associated with lower respiratory tract infection, moxifloxacin was associated with a factor increase in MIC of 4.82 (95% CI 1.44 to 16.19, p=0.01), doxycycline 3·74 (95% CI 1.46 to 9.58, p=0.01) and azithromycin 6.23 (95% CI 1.66 to 23.35, p=0.01) for isolates cultured in sputum taken from patients assigned to those antibiotics compared with placebo. Modelling the number of resistant isolates (where MIC break points were available for specific isolates to specific antibiotics), isolates from patients in the doxycycline group were more likely to be resistant to doxycycline than placebo (OR 5.77 (95% CI 1.40 to 23.74, p=0.02)). ORs for the other antibiotics were also >2 but these were not statistically significant. Boxplots demonstrating the mean MIC concentrations are shown in figure 3.

#### Lung function

There was a mean increase in FEV_1_ compared with placebo of 40 mL with moxifloxacin, 30 mL with doxycycline and 0 mL with azithromycin, although these changes were not significant at the 5% level, even after further adjustment (table 2).

#### Health status

The changes in SGRQ total score are listed in table 2. After adjustment, the largest improvement was seen with azithromycin compared with placebo, but these changes were again not significant at the 5% level.

#### Adherence to therapy

Adherence to therapy was high in all treatment arms (mean (SD) adherence 95%,^13^ 93%,^20^ 96%^11^ and 95%^9^ for moxifloxacin, doxycycline, azithromycin and placebo, respectively). No significant differences were detected between groups (table 2).

#### Exacerbations

**Figure 4:**
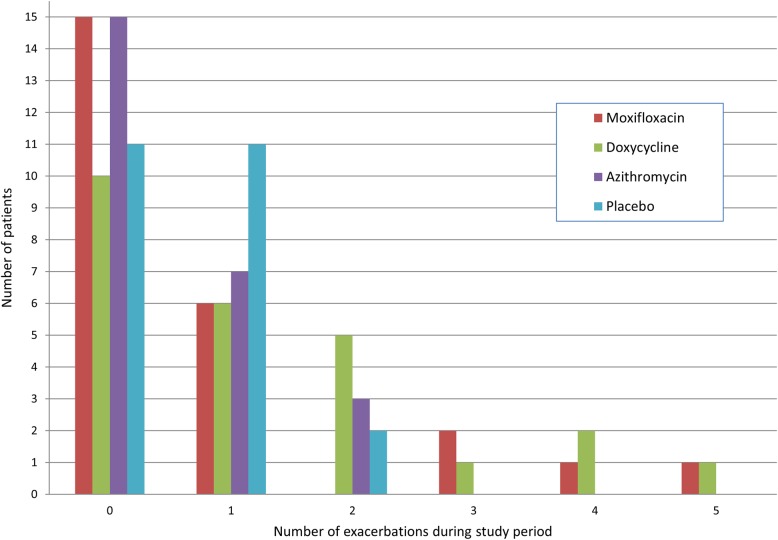
Frequency of exacerbations experienced by patients during the study period, by treatment group.

During the study, 41 patients experienced 81 distinct exacerbations (21 on moxifloxacin, 32 on doxycycline, 13 on azithromycin and 15 on placebo). The relative risks of exacerbation are shown in table 2; there was an indication of increased exacerbations in the doxycycline group, although there was a small number of patients in the doxycycline and moxifloxacin groups who reported frequent exacerbations during the study period. Figure 4 shows the number of exacerbations by treatment group.

#### Adverse events

The highest number of treatment-related adverse events, 10 (40%), was reported with moxifloxacin therapy. These were predominantly minor; therapy was withdrawn in four cases. There were two treatment-related adverse events reported with doxycycline and one with azithromycin. There were no treatment-related serious adverse events or suspected unexpected serious adverse reactions. Further detail is contained in the supplementary data.

#### Comparison of airway load measurements using quantitative culture and 16S qPCR

**Figure 5:**
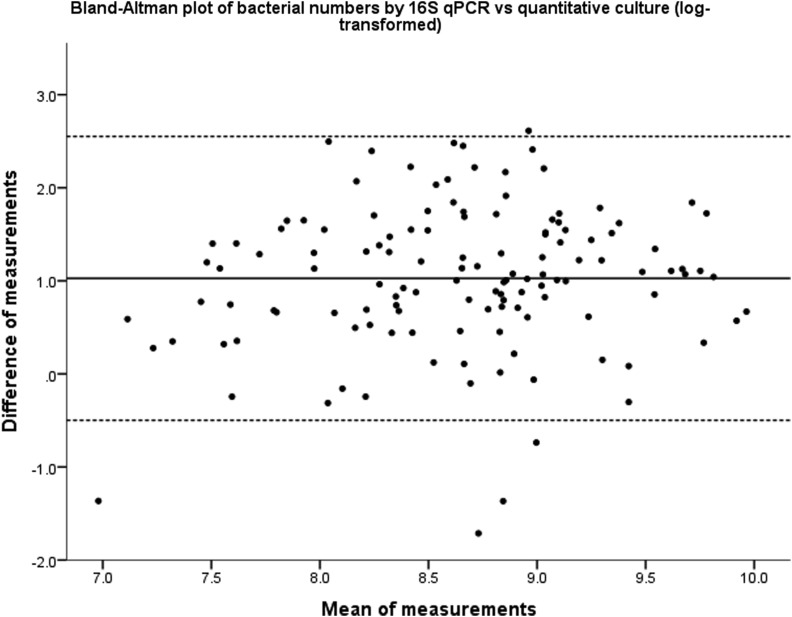
Bland–Altman plot showing the differences between the measurement techniques of quantitative culture and 16S quantitative PCR (qPCR). The solid line is the mean measurement distance and dotted lines are mean±1.96 (SD), that is, the values between which 95% of the measurement differences lie.

Across all samples measured by both methods, 16S qPCR detected 10-fold more bacteria than quantitative culture (excluding samples where no organisms were detected on culture, n=13) (mean (SD) 9.15 (0.79) log_10 _copies/mL vs 8.12 (0.71) log_10_ cfu/mL, p<0.001). There was a significant correlation between the two techniques (Pearson’s r=0.465, p<0.001). Comparison of the measurement techniques using a Bland–Altman plot showed a 3.5-log variation in the measurement differences, although there was no proportional bias across the measured range of values (figure 5).

## Discussion

This study, for the first time, reports on the direct comparison of 13 weeks of therapy using different antibiotic classes and different measurement techniques against the endpoint of airway bacterial load in stable COPD. Most notably, there were no significant reductions in sputum bacterial load, by culture or qPCR, with any of these three antibiotics compared with placebo. Although pulsed moxifloxacin demonstrated the largest fall in bacterial numbers on culture, it also had the highest incidence of associated adverse events. There was evidence of an increase in antibiotic resistance of the cultured isolates across all treatment groups.

The primary endpoint of this study was total bacterial load, as the sum of the load of all isolates from each sample. Most of the bacteria isolated here were species classically considered to be non-pathogenic in airway disease, and this is in keeping with molecular studies of the airway microbiome which demonstrate a diverse flora dominated by firmicutes, including the *Streptococcus* and *Rothia* spp predominant here.^20^ While previous exacerbation studies have focused on loads of the three typical respiratory tract bacteria (*S. pneumoniae, Haemophilus influenzae or Moraxella catarrhalis*),^21^ ^22^ which appear to increase at exacerbation,^21^ more recent investigation of the overall airway microbiome during naturally occurring exacerbations^23^ did not find an increase in total bacterial numbers. As in the gut, the lung microbiome is commensal and plays a role in immune function;^24^ these non-pathogenic microorganisms are therefore also important, and changes in their numbers are likely to influence exacerbation susceptibility. We did not find any support for our hypothesis that antibiotic therapy reduces total bacterial load in stable COPD, as well as potentially improving clinical outcome measures. Airway inflammation also did not change during treatment with any of these antibiotics and this is in keeping with previous reports^9^ and despite known anti-inflammatory effects of macrolide antibiotics. As discussed below, patients in the doxycycline and moxifloxacin arms suffered more exacerbations during the study period, and given that bacterial load,^21^ airway inflammation^25^ and quality of life^1^ all worsen significantly at exacerbation this may have attenuated any measured reductions. In addition, reductions in total bacterial load may only persist for a short time after quinolone therapy^26^ which might reduce the effectiveness of intermittent, ‘pulsed’ dosing regimens.^12^

Overall, however, antibiotic therapy did not appear to reduce total bacterial load after 3 months of therapy when measured either by culture or by 16S qPCR. The reduction in exacerbation frequency observed elsewhere is therefore likely to be mediated by other mechanisms. These may include either altering the background composition of the airway microbiome such that exacerbation susceptibility is reduced, by making the airway a less hospitable environment for the acquisition of new bacterial strains that may potentially cause exacerbation^27^ or alternatively by attenuating the rise in bacterial load when an exacerbation occurs rather than reducing it in the stable state. Further research is required to investigate these possibilities.

For the first time, we have also directly analysed the measurement techniques of 16S qPCR and quantitative culture in the quantification of the airway microbiome. qPCR detected approximately 10 times as many bacteria as culture and, although significantly correlated, the wide variation between the measured values limits their direct comparability. Culture-based techniques are less sensitive than qPCR, and a normally cultivable species may not grow on culture media even when it is proved on sequencing to be the dominant organism in a sample.^20^ These findings therefore support the move away from culture-based to molecular techniques.

Administration of each antibiotic was associated with an MIC increase of at least three times pretreatment values compared with placebo, with a corresponding increase in clinical resistance in those organisms for which this has been defined; this supports findings from other short-term studies of antibiotic therapy.^28^ However, in contrast to previous studies of antibiotics in COPD focusing on resistance in specific pathogens,^10^ ^12^ we examined all isolates cultured from sputum, and we have examined resistance in greater detail. The information here therefore provides a much better indication of resistance in the wider airway microbiome. Macrolide resistance is rapidly inducible, highly transferrable, and increasing in prevalence commensurate with increasing macrolide prescriptions;^13^ resistance to quinolones and tetracyclines is also increasing globally.^29^ ^30^ There is evidence that commensal airway streptococci act as a reservoir for *S. pneumoniae* resistance^31^ and the increased resistance demonstrated here may therefore drive future antibiotic-resistant disease. The approach of only examining resistance in those species classically thought to be pathogenic may be too narrow.

There was an indication of a higher rate of treatment-related adverse events and treatment discontinuation with moxifloxacin therapy than previously reported,^12^ although the types of events reported were similar. This may be related to the higher frequency of administration here (4-weekly rather than 8-weekly) than in the previous study. By contrast, azithromycin and doxycycline were well tolerated. However, there is growing evidence of increased cardiovascular risk with macrolides,^11^ ^32^ as well as high-frequency hearing loss,^10^ although the size and duration of this trial were not sufficient to examine these. Until these risks are further evaluated, advice to exercise caution in patients with cardiac risk factors^33^ still seems prudent.

There were no significant changes seen in the other measures of quality of life, lung function or adherence to therapy. There was an apparent increase in exacerbations with doxycycline therapy, although this was influenced by a small number of patients, and the study period was relatively short for exacerbation detection. Although the placebo group had a lower self-reported exacerbation frequency than the active treatment arms, this was adjusted for in the analysis. Furthermore our composite detection of exacerbations (using diary cards and/or treatment) may have contributed to the relatively higher recorded exacerbation frequency during the study. This study was not powered to detect changes in exacerbation frequency and this result should therefore be treated with caution.

There were a few limitations to this study. First, due to difficulty in double-blinding each of the three treatment regimens in a single trial, only one placebo was used and the trial was single blind. The final sample size was 50% lower than that initially specified (99 compared with 200), and some patients were unable to spontaneously expectorate sputum at their final study visit. This study was intentionally designed as a pilot exploratory study to assess airway bacterial changes with different antibiotics prior to a definitive study. Hence, the precision of the estimates shown here cannot fully discount a reduction in airway bacterial numbers of up to 0.9 log_10_ cfu/mL for moxifloxacin and 0.5 log_10_ cfu/mL for doxycycline and azithromycin. Nevertheless, we have shown that larger reductions in airway bacterial numbers (>1.0 log_10_, or 10-fold, cfu/mL) are unlikely. While the study was terminated early due to constraints with time lines, further recruitment would therefore have been unlikely to alter these conclusions. Third, as an exploratory study, this trial was not powered to detect changes in clinical endpoints and therefore further trials will be needed regarding the relative effectiveness of these antibiotics in practice.

In summary, we found no significant changes in total bacterial load with antibiotic therapy in this, the first randomised, blinded, placebo-controlled study to directly compare the use of different antibiotics in stable COPD. We have also demonstrated an increase in the degree and rate of resistance of cultured airway bacteria following administration of these antibiotics.

## Supplementary Material

Web supplement
