# Supplementary material for: Effects of different antibiotic classes on airway bacteria in stable COPD using culture and molecular techniques: a randomised controlled trial
Source: Thorax. 2015 Jul 15;70(10):930–8. doi: 10.1136/thoraxjnl-2015-207194 (PMC4602260; doi:10.1136/thoraxjnl-2015-207194)
Supplement: Web supplement [file thoraxjnl-2015-207194-s1.pdf]

SUPPLEMENTARY DATA

To accompany Brill, SE et al., ‘Effects of different antibiotic classes on airway bacteria in stable COPD using culture and molecular techniques: a randomised controlled trial/

1. Inclusion and exclusion criteria

Patients were included if they fulfilled the following criteria:

1) Age ≥45 years at screening.
2) COPD confirmed at screening, as defined by forced expiratory volume in one second (FEV_1) <80% predicted with FEV_1/forced vital capacity (FVC) ratio <0.7 and a history of smoking or other plausible irritant exposure.
3) Chronic sputum production, defined as expectoration of sputum on most days; patients were not randomised until a spontaneously expectorated sample had been collected.
4) Able to give informed consent and able to complete symptom questionnaires and a daily diary card.

Principal exclusion criteria were as follows:

1) Other clinically significant respiratory disease
2) Exacerbation of COPD in the four weeks preceding screening or before randomisation
3) Safety criteria
    a. Clinically significant hepatic or renal impairment on screening blood tests.
    b. Evidence of tuberculosis on screening sputum sample given at recruitment.
    c. Uncontrolled hypertension
    d. Prolonged Q-T interval on screening electrocardiogram (ECG) or a history of long QT syndrome
    e. Patients already taking long term antibiotics for any reason or any other contraindicated medication.
    f. Hypersensitivity to any of the antibiotics under evaluation

2. Further information about recruitment procedures.

Primary care recruitment:

Local general practices and pulmonary rehabilitation groups were asked to identify patients on their COPD databases and to send out invitation letters containing the patient information sheet and a reply slip and envelope pre-addressed to the study team. Interested patients replied directly to the study team and were invited for screening. In all, approximately 2750 letters were sent with a reply rate of approximately one fifth. Only 7%

of patients to whom letters were sent ultimately attended for screening. 62 of the 99 enrolled patients were recruited via this method.

In addition to this, members of the study team (SEB and JPA) also attended meetings of pulmonary rehabilitation and patient support groups to deliver education sessions and disseminate information directly about the study. Interested patients were invited for screening. 15 of the 99 enrolled patients came from these sessions. The remaining 22 enrolled patients were identified through hospital outpatient clinics and local research cohorts.

We have reported further information regarding recruitment of COPD patients from primary care elsewhere (Brill, SE et al. Community-based recruitment of patients with COPD into clinical research. Thorax. 2014 Mar 4. doi: 10.1136/thoraxjnl-2014-205253).

3. Sputum processing

Quantitative culture

Spontaneously expectorated sputum samples were collected from patients and processed within six hours. Sputum plugs were separated from contaminating saliva by macroscopic examination using sterile forceps and homogenised using Sputasol (Oxoid Ltd, Basingstoke, UK) and serial dilutions prepared. 10µl of each dilution was inoculated onto agar plates and spread with a sterile 'hockey stick'. After incubation in 5% CO_2 for 24 hours, viable bacteria were counted from those organisms that exhibited between 30-300 discrete colonies and the results expressed as cfu/ml after correcting for dilution factor and sample volume. The sum of the loads for all isolates was expressed as the total bacterial load. At the time of culture, isolates were stored on glass beads at -80°C for future use. Any remaining sputum after culture was homogenised with isotonic phosphate buffered saline and stored at -80°C pending qPCR and inflammatory marker analysis.

Antibiotic Resistance Testing

In order to test for antibiotic resistance, isolates were subsequently thawed in batches. After spreading onto agar plates, prepared antibiotic gradient strips (Etest®, bioMérieux UK Ltd, Basingstoke, UK) were used to determine the main inhibitory concentrations in accordance with the manufacturer's instructions.

Quantitative Polymerase Chain Reaction (qPCR) for 16S bacterial ribosomal RNA gene

DNA was isolated from sputum using the FastDNA™ SPIN Kit for Soil (MP Biomedicals, Santa Ana, CA. 1:10 standard dilutions were prepared using a working solution of *Pseudomonas Aeruginosa* PA01 16S rRNA gene. PCR reactions were carried out in triplicate, each well containing 7.5µl SYBR Fast qPCR Master Mix (KAPA Biosystems, London, UK), 0.3µl 10µM forward (520F: 5'- AYT GGG YDT AAA GNG -3') and 0.3µl 10µM (802R: 5' TAC NVG GGT ATC TAA TCC -3') reverse primers (Eurofins MWG Operon, Ebersberg, Germany), 1.9 µl PCR

water and 5µl of either sample (diluted 1 in 5µl), non-template control or standard. Data was acquired using a ViiA 7 real-time PCR system (Life Technologies, Paisley, UK) with cycle conditions of 90°C for 3 minutes then 40 cycles of 95°C for 20 seconds, 50°C for 30 seconds and 72°C for 30 seconds. 16S copy numbers per gram of sputum were extrapolated from the cycle threshold for each sample using ViiA7 Software Base v1.1 (Life Technologies, Paisley, UK).

4. Further information about statistical analysis of secondary endpoints

Quality of life was analysed using the St George's Respiratory Questionnaire, a well validated measure of health status/. The questionnaire contains 50 items, with 76 graded responses, and an overall score is returned from 0-100 where higher scores indicate worsened health status.

Adherence was defined as (pills taken) / (pills expected to be taken), while (pills taken) was defined as (pills given) – (pills returned).

Exacerbation frequency was analysed using negative binomial regression, accounting for each individual's time at risk.

Antibiotic resistance was modelled using a linear mixed effects model for log(MIC) as a continuous variable. In order to model resistance as a binary outcome, MIC break points were defined where available from the European Committee on Antimicrobial Sensitivity Testing (EuCAST, www.eucast.org), British Society for Antimicrobial Chemotherapy (bsac.org.uk), Clinical Laboratory Standards Institute (clsi.org) and the Etest manufacturer's guidelines. Isolates were classified as resistant, sensitive, or intermediate according to whether the measured MIC was above, in, or below the breakpoint range. Odds ratios of resistance were analysed using a generalised mixed effects model. Analyses reverted to using multiple regression or logistic regression when the between isolate variance was estimated to be zero or when the mixed effects model failed to converge.

5. Further results: Adverse events

Adverse events table:

Moxifloxacin	Doxycycline	Azithromycin	Placebo
Nausea +/- vomiting: 5<br>Rash: 2<br>Suspected Achilles tendonitis: 1<br>Other: 2	Nausea +/- vomiting: 2	Flatulence: 1	
